# Peculiarities of Dentition and Dental Disease Hereditary

**Published:** 1867-06

**Authors:** 


*Peculiarities of Dentition and Dental disease Hereditary.*—In a paper by William Sedgwick, in the April number of the *British and Foreign Medico-Chirurgical Review*, we find some interesting statements in reference to this subject.

The author cites Dr. Montgomery's case of the two central incisors of the lower jaw projecting from the gum at birth in two children of the same mother. Dr. Whitehead records a similar peculiarity, two children and their mother having all three been born with the two lower incisors fully developed. Retardation, however, is more common. The author mentions a case, in his own experience, in which a little girl, aged fourteen months had not cut a tooth. The peculiarity was traced to a paternal grandmother, who, as well as her seven children, had not begun to cut teeth before attaining the fifteenth month. Four of these seven children, (three males and one female) grew up and married, and had amongst them five children, all of whom delayed cutting their teeth until after the first year.

A similar hereditary law governs occasionally the developement of caries. This disease attacked the upper incisors of five children in the same family, commencing at the age of five years. The first born was a girl whose upper central incisors began to decay at the age of two years. Corresponding decay of the same teeth, at the same age, occurred in the second, third fifth and sixth children. The fourth child died at the age of fifteen months, too early for the developement of the disease, and the seventh was an infant four months old when the paper was written. This is certainly a very remarkable history, and it is impossible to avoid the conclusion, that the children inherited same sort of defect of these particular teeth, although their parents had sound front teeth, and no similar early caries was known to have occurred in the family.

